# In Vivo Toxicity Assessment of the Probiotic *Bacillus amyloliquefaciens* HTI-19 Isolated from Stingless Bee (*Heterotrigona itama)* Honey

**DOI:** 10.3390/nu15102390

**Published:** 2023-05-19

**Authors:** Fatin Aina Zulkhairi Amin, Mohamad Zulhafiz Shafiq Cheng, Suriana Sabri, Norsharina Ismail, Kim Wei Chan, Norhaizan Mohd Esa, Mohd Azmi Mohd Lila, Saulol Hamid Nur-Fazila, Shaden A. M. Khalifa, Hesham R. El-Seedi, Norhasnida Zawawi

**Affiliations:** 1 Natural Medicines and Product Research Laboratory, Institute of Bioscience, Universiti Putra Malaysia, Serdang 43400, Selangor, Malaysia; fatinainaza@gmail.com (F.A.Z.A.); zulhazshafiq@gmail.com (M.Z.S.C.); norsharina@upm.edu.my (N.I.); chankim@upm.edu.my (K.W.C.); nhaizan@upm.edu.my (N.M.E.); 2 Enzyme and Microbial Technology Research Center, Faculty of Biotechnology and Biomolecular Sciences, Universiti Putra Malaysia, Serdang 43400, Selangor, Malaysia; suriana@upm.edu.my; 3 Department of Microbiology, Faculty of Biotechnology and Biomolecular Sciences, Universiti Putra Malaysia, Serdang 43400, Selangor, Malaysia; 4 Department of Nutrition and Dietetics, Faculty of Medicine and Health Sciences, Universiti Putra Malaysia, Serdang 43400, Selangor, Malaysia; 5 Department of Veterinary Pathology and Microbiology, Faculty of Veterinary Medicine, Universiti Putra Malaysia, Serdang 43400, Selangor, Malaysia; azmi@upm.edu.my (M.A.M.L.); nurfazila@upm.edu.my (S.H.N.-F.); 6 Department of Molecular Biosciences, Wenner-Gren Institute, Stockholm University, SE-106 91 Stockholm, Sweden; shaden.khalifa.2014@gmail.com; 7 International Research Center for Food Nutrition and Safety, Jiangsu University, Zhenjiang 212013, China; hesham.el-seedi@farmbio.uu.se; 8 International Joint Research Laboratory of Intelligent Agriculture and Agri-Products Processing, Jiangsu Education Department, Jiangsu University, Zhenjiang 212013, China; 9 Functional Carbohydrate and Protein Laboratory, Faculty of Food Science and Technology, Universiti Putra Malaysia, Serdang 43400, Selangor, Malaysia; 10 Laboratory of Halal Science, Halal Products Research Institute, Universiti Putra Malaysia, Serdang 43400, Selangor, Malaysia

**Keywords:** functional foods, probiotic safety, Sprague Dawley rat

## Abstract

This study evaluated the acute and sub-acute toxicity of *B. amyloliquefaciens* HTI-19 (isolated from stingless bee honey) in female Sprague Dawley rats. In an acute toxicity study, the rats received a low dosage (1 × 10^9^ CFU·mL^−1^), medium dosage (3 × 10^9^ CFU·mL^−1^), or high dosage (1 × 10^10^ CFU·mL^−1^) of *B. amyloliquefaciens* HTI-19 daily orally by syringe-feeding for 14 days. For the subacute toxicity study, rats received a low dosage (1 × 10^9^ CFU·mL^−1^) or a high dosage (1 × 10^10^ CFU·mL^−1^) for 28 days. The probiotic feeding in acute and sub-acute toxicity studies showed no mortality or significant abnormalities in rats throughout the experimental period. In week 2 of the acute study, the body weight of the rats showed a significant increase (*p* < 0.05) compared to the control. By gross and microscopic examination of organs, no evidently significant changes were observed in the morphology of organs. Serum biochemical tests and blood hematology tests also revealed no treatment-related changes. Overall, these data indicated that oral administration of *B. amyloliquefaciens* HTI-19 up to 1 × 10^9^ CFU·mL^−1^ for 28 days can be considered safe.

## 1. Introduction

Probiotics have been effective in treating a wide variety of illnesses and infections [1], [2]. Considering their medicinal attributes, the widespread use of probiotic bacteria has not been controlled in terms of safety and toxicity at different doses or routes of administration [3]. Beneficial probiotics are known to have no infectivity, pathogenicity and do not cause side effects or display toxicological properties [4,5].

*Bacillus* species are some of the commercialized probiotics, alongside the more well-known probiotic genera of *Lactobacillus* and *Bifidobacterium* [6]. *Bacillus* unlike these two genera, does not hold the “Generally Recognized as Safe” (GRAS) status, since some *Bacillus* are identified to be pathogenic. Thus, it is vital to assess its safety in order to investigate its impact on human health and human ingestion. Several studies have evaluated the toxicity of *Bacillus* sp., mainly *B. subtilis, B. coagulans, B. licheniformis*, and *B. clausii,* in vivo [7] and recently on *B. amyloliquefaciens* [8], where no adverse effects were observed on the tested animals. However, probiotic benefits and pathogenicity are strain-specific [9]. Thus, strain toxicity should be evaluated routinely and individually. 

Stingless bee honey (SBH) is known by many other different names, such as Meliponine honey, pot-honey, and Kelulut honey (in Malaysia) [10,11]. The stingless bee, *Heterotrigona itama,* is the dominant species inhabiting Malaysia [12]. Previously, we have demonstrated the in vitro probiotic potential of *Bacillus* strains isolated from SBH. *Bacillus amyloliquefaciens* strain HTI-19 demonstrated the best probiotic characteristics with good antimicrobial activity, high survival in acid and bile environments, and adhesion to hydrocarbons [13]. Although we found *B. amyloliquefaciens HTI-19* to show α-hemolytic activity (partial red blood cell breakdown), an in vivo test is more appropriate to study its virulence [14]. Honey, in general, is a reservoir for beneficial microorganisms, including probiotic bacteria [9,15]. In addition, it has been known for its nutritious and therapeutic qualities since the 20th century due to its exceptional antibacterial, bacteriostatic, anti-inflammatory, wound, and sunburn healing effects [16]. Moreover, the honey’s chemical and biological properties have been intensively reported [17].

This research was conducted to assess the toxicity of *B. amyloliquefaciens* HTI-19 isolated from stingless bee (*Heterotrigona itama*) honey using acute and subacute oral toxicity tests in rats as part of our ongoing project on honeybees and bee products [18,19,20,21]. 

## 2. Materials and Methods

### 2.1. Animal Study

The use of animals in this study was approved by the Animal Care and Use Committee, Faculty of Medicine and Health Sciences, Universiti Putra Malaysia (Project approval number UPM/IACUC/AUP-R008/2019). In an acute and subacute toxicity study, 9–10-week-old female Sprague Dawley rats were obtained from A Sapphire Enterprise, Selangor, Malaysia, and were subjected to 7 days of acclimatization. Free access to food and water was provided throughout the study period. Experimental procedures and handling were performed according to the animals’ guidelines [22]. 

### 2.2. Preparation of B. Amyloliquefaciens HTI-19 Culture

*Bacillus amyloliquefaciens* HTI-19 used in this study was previously isolated from stingless bee *(Heterotrigona itama)* honey [13]. The strain was cultured in a nutrient broth medium (Oxoid, UK) and incubated for 24 h at 37 °C in a shaker incubator. After incubation, the culture was centrifuged at 5000× *g* for 10 min at 4 °C. Supernatants were discarded, and cell pellets were washed three times and resuspended in phosphate-buffered saline (PBS), pH 7.4. The concentration of bacteria was standardized at OD 7.4, 8.7, and 10.2 (600 nm) using a UV–VIS spectrophotometer (UV-1800, Shimadzu, Japan) to give viable counts of approximately 1 × 10^9^ CFU·mL^−1^, 3 × 10^9^ CFU·mL^−1^ and 1 × 10^10^ CFU·mL^−1^, respectively. The culture pellets were stored in 20% glycerol and kept at −20 °C until further use. Cell suspensions were freshly prepared from the glycerol stocks every day before feeding the animals via syringe feeding. After centrifugation (5417, Eppendorf, Hamburg, Germany) at 5000× *g* for 10 min at 4 °C, the pellet was resuspended in the same 10% UHT milk volume. 

### 2.3. Acute Oral Toxicity Study

A 14-day acute oral toxicity study (repeated dose) was performed according to the OECD Guideline for the Testing of Chemicals No. 425, Acute Oral Toxicity Up and Down Procedure, adopted on 17th December 2001 [22]. A total of 32 female rats were used. After 7 days of acclimatization and 16 h of fasting, rats were randomly divided into four groups (*n* = 8), and different treatments were given to each group of animals (Table 1). Each treatment was administered (by syringe-feeding) once daily for 14 consecutive days.

### 2.4. Sub-Acute Oral Toxicity Study

A 28-day sub-acute oral toxicity study (repeated-dose) was performed according to the OECD Guideline for the Testing of Chemicals No. 407, Repeated Dose 28-Day Oral Toxicity Study in Rodents, adopted on 3rd October 2008 [23]. A total of 24 female rats were used. After seven days of acclimatization and a 16 h fasting period, the rats were randomly divided into three groups (*n* = 8): each group of animals received one of the treatments (Table 2).

### 2.5. General Observations

The general observations for the oral toxicity study include any changes to the skin and fur, respiratory activity, movement activity, behavior pattern, tremors, convulsions, salivation, diarrhea, fatigue, sleep pattern, and posture changes [22]. Physical parameters observed during the experimental period are bodyweight, local injuries, and death (if any).

### 2.6. Relative Organ Weights and Histopathological Analysis

Following sacrification, rats’ hearts, lungs, liver, spleen, and kidneys were harvested, and gross pathological observations such as organ weights, macroscopic appearance, and presence of lesions were carried out for all organs. The calculation of the relative weight for each organ was as follows:

(1) $$\mathrm{The}\;\mathrm{relative}\;\mathrm{weight}\;\mathrm{of}\;\mathrm{the}\;\mathrm{organ},\;\left(\%\right)=\frac{\mathrm{weight}\;\mathrm{of}\;\mathrm{organ}}{\;\mathrm{live}\;\mathrm{bodyweight}}\times 100$$

Histopathological examinations were performed on rats’ hearts, lungs, liver, spleen, and kidneys in the subacute oral toxicity study [24]. The preservation of tissues and organs was performed in 10% formalin before further analysis. Prior to use, it was dehydrated for 16 h in an automatic tissue processor (Leica ASP 3000, Tokyo, Japan), and using a paraffin embedding system (Leica EG 1160, Tokyo, Japan), the tissues were embedded in paraffin wax. A section of 4 μm thickness of each sample was cut using a rotary microtome (Leica RM 2155, Tokyo, Japan). The sections were then placed on a glass slide and fix-heated until dried at 57 °C. Haematoxylin and eosin were used to stain the sample for examination under a light microscope (Dialux, Leitz Wetzlar, Germany).

### 2.7. Serum Biochemistry and Hematological Analyses

Rats were anaesthetized with ketamine-xylazine at the end of the experiment, and blood samples were collected via cardiac puncture for hematology and serum biochemistry analyses. Blood serum for biochemistry analysis was collected in non-heparinized tubes. Collected blood was left at room temperature for a while to allow clotting before serum collection by centrifugation (3000× *g* for 10 min) at 4 °C. The collected serum was stored at −4 °C before analysis using an automatic clinical chemistry analyzer (Hitachi, Japan). The parameters analyzed for serum biochemistry analysis were as follows: albumin, aspartate aminotransferase, alanine aminotransferase, alkaline phosphatase, creatinine, total bilirubin, urea, and total protein. 

Blood was collected in K2EDTA tubes using an automated hematology analyzer (CELL-- DYN 3700 Abbott Diagnostics, Des Plaines, IL, USA). Parameters analyzed for whole blood cells were as follows: red blood cell count, hemoglobin, packed cell volume, mean corpuscular volume, mean corpuscular hemoglobin concentration, thrombocytes, and white blood cell count (including lymphocytes, monocytes, neutrophils, and eosinophils) [24].

### 2.8. Statistical Analysis

Experimental data were analyzed by a one-way ANOVA procedure using GraphPad Prism 6.0 (GraphPad Software, Inc., San Diego, CA, USA). Differences were considered significant if *p* < 0.05. Multiple comparisons among the significant means were performed using Dunnett’s test.

## 3. Results

### 3.1. Acute and Sub-Acute Toxicity of B. Amyloliquefaciens HTI-19

For the acute toxicity study, *Bacillus amyloliquefaciens* HTI-19 was administered by syringe feeding at different doses of 1 × 10^9^ CFU·mL^−1^ (low dose), 3.0 × 10^9^ CFU·mL^−1^ (medium dose), and 1 × 10^10^ CFU·mL^−1^ (high dose) for 14 consecutive days. The acute toxicity test showed no lethality or toxicity throughout the experimental period. For the subacute toxicity study, *B. amyloliquefaciens* HTI-19 culture was administered by syringe feeding at 1 × 10^9^ CFU·mL^−1^ (low dose) and 1 × 10^10^ CFU·mL^−1^ (high dose) for 28 consecutive days. There were no adverse effects or treatment-related signs of toxicity based on clinical observation. No gross pathological changes were found in rats of all groups. 

### 3.2. Effects of Oral Administration of B. Amyloliquefaciens HTI-19 on Body Weight

**Figure 1:**
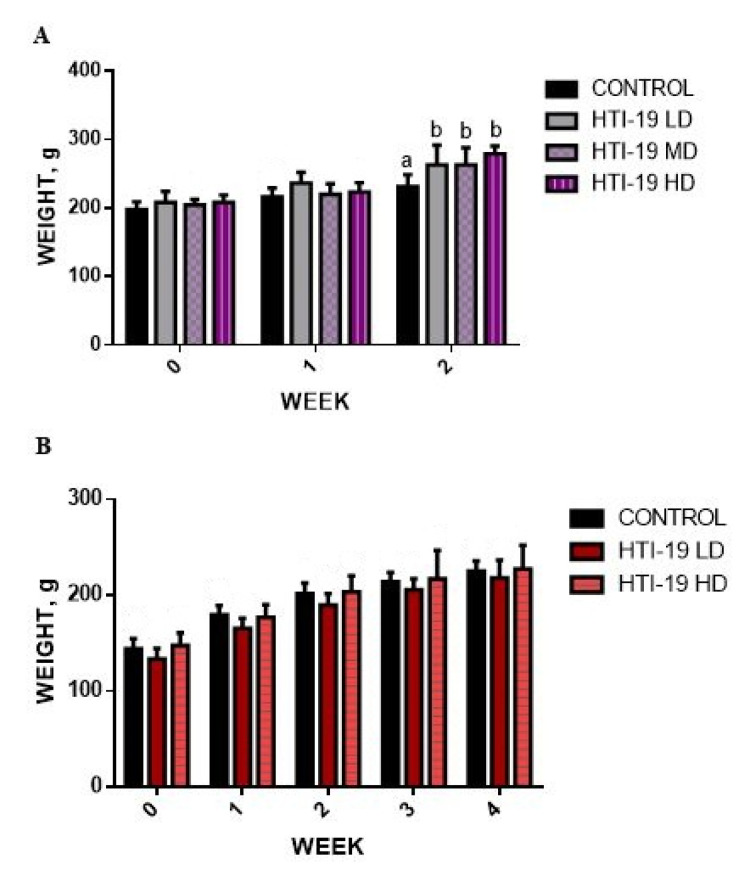
Effects of *B. amyloliqiefaciens* HTI-19 on body weight in (**A**) an acute oral toxicity study and (**B**) a sub-acute oral toxicity study**.** ^a,b^: Different letters in the same vertical column indicate statistical differences in each strain at the level of *p* < 0.05. Values are expressed as mean ± S.D. (*n* = 8). Statistical analysis was performed by one-way ANOVA followed by Dunnet’s t-test. (LD-low dose, MD-medium dose, HD-high dose).

In the acute study, the body weight of rats was not affected by the daily administration of probiotic *B. amyloliquefaciens* HTI-19 from weeks 0 to 1 (Figure 1a). However, in week 2, the body weight of the rats receiving *B. amyloliquefaciens* HTI-19 started to show a significant increase (*p* < 0.05) compared to the control. In contrast, the rats’ body weights in all treatment groups of the subacute toxicity study were not significantly different (*p* > 0.05) from those of rats in the control groups throughout the experimental period (Figure 1b). 

### 3.3. Effects of Oral Administration of B. Amyloliquefaciens HTI-19 on Relative Organ Weights and Histopathological Examination

The relative organ weights for the lung, heart, right kidney, left kidney, liver, and spleen showed no significant difference (*p* > 0.05) between the control and all treatment groups in the acute toxicity study (Table 3). Following the autopsy, no noticeable pathological changes were observed for the selected organs in all treated and control rats. Therefore, further histopathological analyses of rat organs from the acute study were not carried out as stated in OECD Guideline No. 423.

Meanwhile, the relative organ weights for the lung, right kidney, left kidney, liver, and spleen showed no significant difference (*p* > 0.05) between the control and low-dose group in the subacute toxicity study as seen in Table 4. There were also no significant differences in the liver and right kidney relative organ weights in the high-dose group compared to the control group after four weeks of treatment. However, the readings for the lung, heart, spleen, and left kidney did show a significant increase between the control and high-dose group (Table 4). 

**Figure 2:**
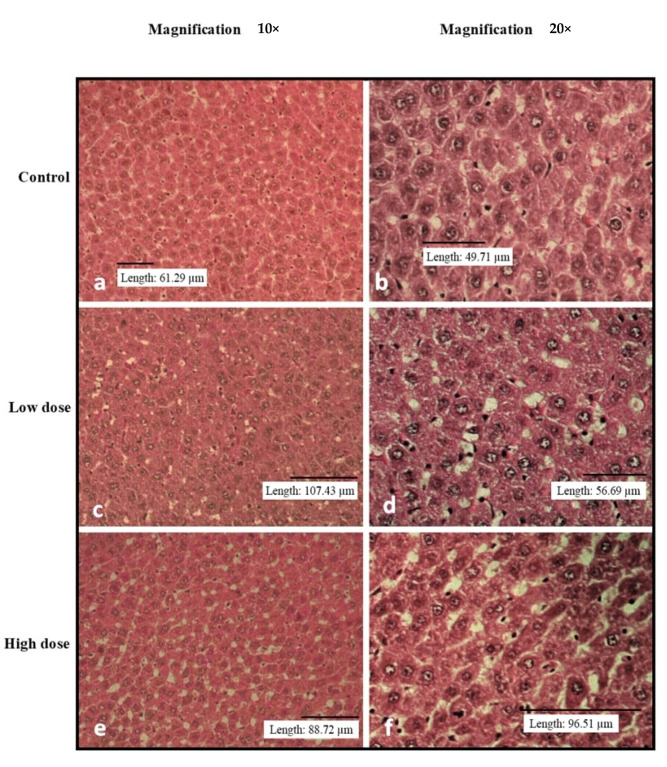
Histopathological analysis of rats’ livers fed with *B. amyloliquefaciens* HTI-19 culture from sub-acute oral toxicity tests. Rats were treated with a low-dose treatment (1 × 10^9^ CFU·mL^−1^ *B. amyloliquefaciens*) and a high-dose treatment (1 × 10^10^ CFU·mL^−1^ *B. amyloliquefaciens*). Microscopic observation showed no significant changes in the histological structure of the liver between the control (**a**,**b**) and treated groups of low dose (**c**,**d**) and high dose (**e**,**f**).

**Figure 3:**
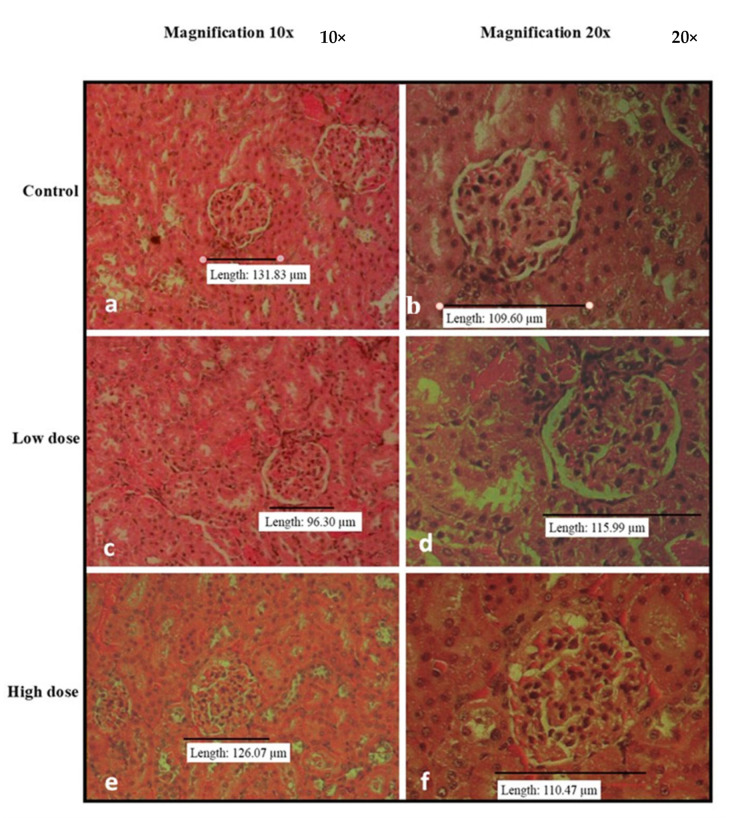
Histopathological analysis of rats’ kidneys fed with *B. amyloliquefaciens* HTI-19 culture from sub-acute oral toxicity test. Rats were treated with low dose treatment (1 × 10^9^ CFU·mL^−1^ *B. amyloliquefaciens*) and high dose treatment (1 × 10^10^ CFU·mL^−1^ *B. amyloliquefaciens*). Microscopic observation showed no significant changes in the histological structure of the kidney between control (**a**,**b**) and treated groups of low dose (**c**,**d**) and high dose (**e**,**f**).

**Figure 4:**
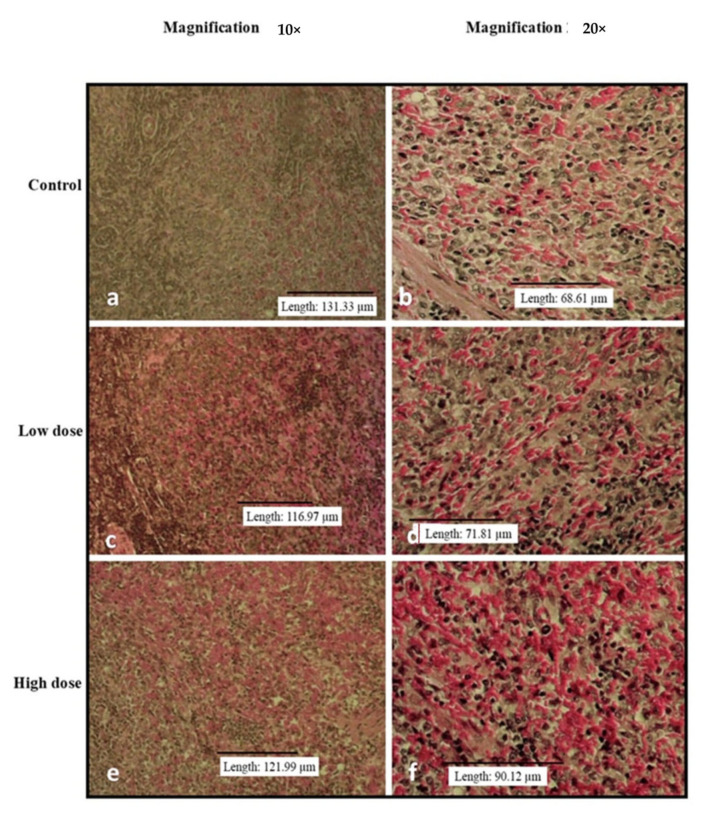
Histopathological analysis of rats’ spleen fed with *B. amyloliquefaciens* HTI-19 culture from sub-acute oral toxicity test. Rats were treated with low dose treatment (1 × 10^9^ CFU·mL^−1^ *B. amyloliquefaciens*) and high dose treatment (1 × 10^10^ CFU·mL^−1^ *B. amyloliquefaciens).* Microscopic observation showed no significant changes in the histological structure of the spleen between the control (**a**,**b**) and treated groups of low dose (**c**,**d**) and high dose (**e**,**f**).

**Figure 5:**
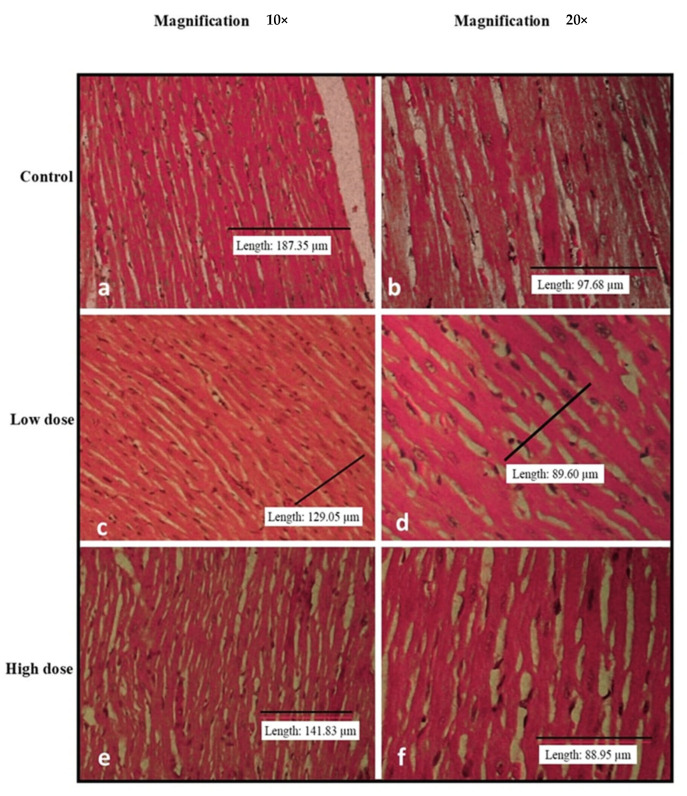
Histopathological analysis of rats’ hearts fed with *B. amyloliquefaciens* HTI-19 culture from the sub-acute oral toxicity test. Rats were treated with low-dose treatment (1 × 10^9^ CFU·mL^−1^ *B. amyloliquefaciens*) and high-dose treatment (1 × 10^10^ CFU·mL^−1^ *B. amyloliquefaciens*). Microscopic observation showed no significant changes in the histological structure of the heart between the control (**a**,**b**) and treated groups of low dose (**c**,**d**) and high dose (**e**,**f**).

**Figure 6:**
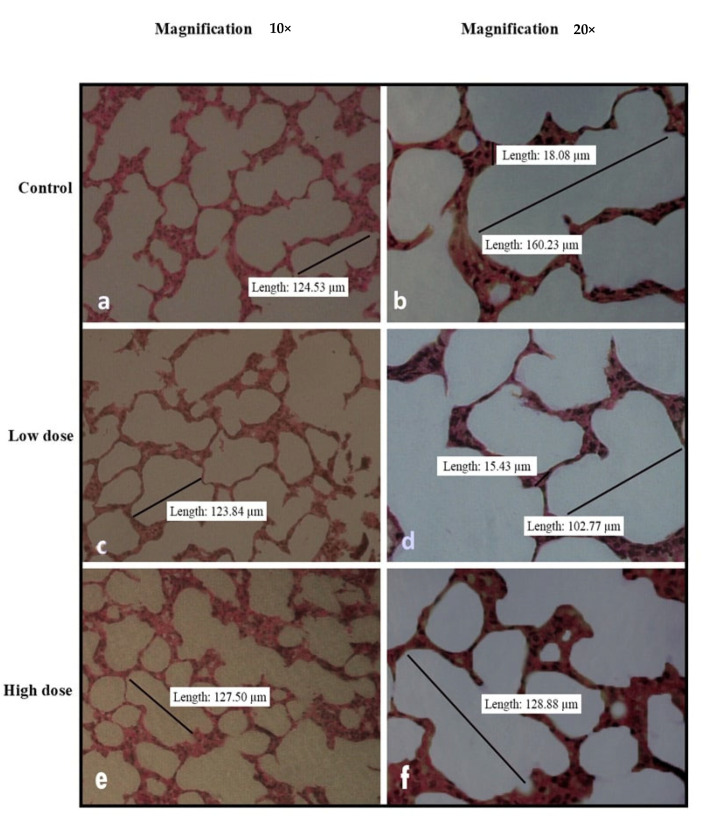
Histopathological analysis of rats’ lungs fed with *B. amyloliquefaciens* HTI-19 culture from the sub-acute oral toxicity test. Rats were treated with low-dose treatment (1 × 10^9^ CFU·mL^−1^ *B. amyloliquefaciens*) and high-dose treatment (1 × 10^10^ CFU·mL^−1^ *B. amyloliquefaciens*). Microscopic observation showed no significant changes in the histological structure of the lung between the control (**a**,**b**) and treated groups of low dose (**c**,**d**) and high dose (**e**,**f**).

Figure 2, Figure 3, Figure 4, Figure 5 and Figure 6 show the microscopic observation of the liver, kidney, heart, lung, and spleen from the subacute oral toxicity test. Overall, there were no obvious significant changes in the observed morphology that could be the result of *B. amyloliquefaciens* HTI-19 administration. The tissues were morphologically normal and similar to those of the treated rats. However, it is noted that the high-dose group’s spleen morphology exhibits higher cellularity than the control group’s (Figure 4e,f).

### 3.4. Effects of Oral Administration of B. Amyloliquefaciens HTI-19 on Biochemical Parameters and Complete Blood Count

Table 5 shows the biochemical parameters in the acute toxicity study. Biochemical tests revealed no treatment-related changes in aspartate transaminase (AST) or alkaline phosphatase (ALT) among the rats in the various groups. Significant decreases were noted in the alkaline phosphatase (ALP), albumin (ALB), total proteins (TP), and creatinine (creat) levels of the high-dose rats in comparison with those of the control groups. 

However, all of these levels are still within the normal range, as reported in other studies [25,26,28]. 

The biochemical parameters in the subacute toxicity study are shown in Table 6. It revealed no significant elevation of the levels of alkaline phosphatase (ALP), aspartate transaminase (AST), alkaline phosphatase (ALT), albumin (ALB), total bilirubin (TBIL), urea, total proteins (TP), and creatinine (creat) in the rats administered with *B. amyloliquefaciens* HTI-19 at different dosages. These data indicated that oral administration of *B. amyloliquefaciens* HTI-19 culture up to 1 × 10^10^ CFU·mL^−1^ for 28 days caused no hepatotoxicity or nephrotoxicity in rats.

Based on the complete blood count depicted in Table 7, there were no significant differences in the numbers of red blood cells (RBC), hemoglobin content, packed cell volume, mean corpuscular volume (MCV), mean corpuscular hemoglobin concentration (MCHC), white blood cell numbers (WBC), monocytes, and eosinophils in the rats administered with *B. amyloliquefaciens* HTI-19 at different doses in comparison with the control, except for neutrophil and lymphocyte counts. Although significantly higher, the values are still within the normal range of neutrophil and lymphocyte counts, as reported by another study [28]. Haematological values were not significantly affected by the administration of *B. amyloliquefaciens* HTI-19 for most of the parameters analyzed.

## 4. Discussion

This study assessed the toxicity of *Bacillus amyloliquefaciens* HTI-19 using acute and sub-acute toxicity tests in rats. Results showed that this strain is safe when orally administered to the treated rats. 

A study by Kotowicz et al. [5] showed a similar result where two *Bacillus* strains, *B. pumilus* and *B. megaterium*, exhibited no symptoms of toxicity and no change in animal behavior after 14 days of oral feeding in the treated rats. Our results also coincide with another toxicity study in Wistar rats using 2 doses of *B. licheniformis* culture (1.1 × 10^10^ and 1.1 × 10^11^ CFU·kg^−1^ body weight, respectively). The dose-dependent acute toxicity and a 90-day subchronic toxicity study resulted in no treatment-related mortality [27].

An increase in the rats’ body weight after the treatment was observed in this study, which was similar to previous data [29], where the putative probiotic *B. amyloliquefaciens* B-1895 was supplemented to fish and *B. amyloliquefaciens* (BA PMC-80) to hamsters [8]. Probiotic addition to animal feed has been associated with an increase in daily weight, thus promoting animals’ health and growth performance [30]. The same findings were also noticed with *B. amyloliquefaciens* TL strains (24) and *B. amyloliquefaciens* HTI-19 in our previous study [13]. In addition, the administration of *B. amyloliquefaciens* in animals also improved nutrient absorption and balanced the intestinal microbiota [31].

Relative organ weight is considered an indicator to detect the degree of drug and chemical toxicity [32]. It is a sensitive parameter of chemically induced organ changes and has been used regularly in toxicity assays. Therefore, the organ weight comparison between control and treated groups has conventionally been tested [33].

In this study, the increased organ weights were compared with the published range of normal rats’ organ weights and were found to be close to the normal weight. Thus, no toxicity effects can be concluded. Other studies have also reported minor changes in the organ weight of the tested animal subjects, which did not corroborate toxicity effects [34,35].

Any damage to hepatic cells will elevate the serum levels of AST, ALT, and ALP enzymes compared to normal conditions [35]. A cirrhotic or fibrotic liver will also increase bilirubin but decrease albumin and total protein levels in the blood. Meanwhile, kidney ailments will increase urea and creatinine levels [36]. In the present study, of acute toxicity tests, ALP, albumin, total proteins, and creatinine levels decreased in the group treated with a high dose. However, all values were still within the normal physiological range [25,26,28]. Therefore, daily administration of *B. amyloliquefaciens* HTI-19 for 14 days was not considered acutely toxic.

The hematopoietic system is a good measure of animals’ and humans’ pathological and physiological states due to its sensitivity to toxic substances. In the current study, no significant differences in the hematological parameters between the treated and control groups were demonstrated. Even though there are significant dose-dependent changes in neutrophil and lymphocyte levels, the values still fall within the normal range [28], confirming that *B. amyloliquefaciens* HTI-19 did not have adverse effects on the circulating blood cells and their production.

These findings suggest that the probiotic *B. amyloliquefaciens* HTI-19 may not be toxic, as the circulating RBC, hematopoiesis, and leucopoiesis were not significantly affected. Another recently reported study using specific pathogen-free (SPF) Kunming mice also reported no signs of toxicity, and, more interestingly, *B. amyloliquefaciens* B10 was found to antagonize oxidative damage and apoptosis induced by aflatoxin B1 (AFB1) in the livers of mice by regulating their intestinal flora [37]. Furthermore, *B. amyloliquefaciens* fmb50 was reported to produce the lipopeptide surfactin, which not only ameliorated high-fat diet and streptozotocin-induced gut dysbiosis and preserved intestinal barrier integrity, but also enhanced hepatic glucose metabolism and detoxification function in type 2 diabetes mellitus mice [38].

Although statistical significance was observed for some parameters in this study (e.g., biochemical parameters, complete blood count, and relative organ weights), we hypothesized that none of these changes were attributable to the treatment as the changes remained within the normal range in comparison to the controls. Fluctuating results were also seen in different feeding groups, as reported earlier [24], [39]. However, these results did not affect the conclusions derived from the safety evaluation.

The results obtained also agreed with the data of *Lactobacillus* [40] and *Bacillus* probiotics [41]. Biochemical results from a 14-day acute study did not show toxicity in rats treated with a high dose (1.0 × 10^10^ CFU·mL^−1^ per rats) of *B. amyloliquefaciens*, corresponding to 70 × 10^10^ CFUs per person of an average 70 kg weight [41]. Thus, the concentration used can be considered 2566 to 77,000 times safe for human consumption, as the suggested human dose is in the range of 1 × 10^8^ to 3 × 10^9^ CFUs. 

## 5. Conclusions

Overall, it can be concluded that the consumption of *B. amyloliquefaciens* HTI-19 was safe until a concentration of 1 × 10^9^ CFU·mL^−1^. It can be regarded as a safe food and has the potential to be utilized in healthy products. Oral administration of *B. amyloliquefaciens* HTI-19 with increasing doses did not produce any mortality or severe abnormalities. No noticeable bodyweight reduction, toxicity symptoms, or death occurred. No obvious gross disruption or distortion of the organs was reported. It is noteworthy that the administration of *B. amyloliquefaciens* HTI-19 promoted the rat’s growth, as seen in the high-dose group compared to the control group. It could promote a healthy intestinal microbial community, preventing drug-resistant microorganisms from spreading. In conclusion, this study further supports the use of *B. amyloliquefaciens* as a potential probiotic as a suitable alternative to antibiotics in prevention and protection strategies directed at food safety. 

## Figures and Tables

**Table 1:** Animal grouping for the acute oral toxicity study.

Animal Grouping	Treatment
Group 1	Normal group (N)—Normal rat, receive a normal diet and 10% UHT milk.
Group 2	Low Dose (LD)—receive a normal diet and probiotic *B. amyloliquefaciens* culture (1 × 10^9^ CFU·mL^−1^) in 10% UHT milk.
Group 3	Medium Dose (LD)—receive a normal diet and probiotic *B. amyloliquefaciens* culture (3 × 10^9^ CFU·mL^−1^) in 10% UHT milk.
Group 4	High Dose (HD)—receive a normal diet and probiotic *B. amyloliquefaciens* culture (1 × 10^10^ CFU·mL^−1^) in 10% UHT milk.

**Table 2:** Animal grouping for the sub-acute oral toxicity study.

Animal Grouping	Treatment
Group 1	Normal group (N)—Normal rat, receive a normal diet and 10% UHT milk.
Group 2	Low Dose (LD)—receive a normal diet and probiotic *B. amyloliquefaciens* culture (1 × 10^9^ CFU·mL^−1^) in 10% UHT milk.
Group 3	High Dose (HD)—receive a normal diet and probiotic *B. amyloliquefaciens* culture (1 × 10^10^ CFU·mL^−1^) in 10% UHT milk.

**Table 3:** Effects of *B. amyloliquefaciens* HTI-19 on relative organ weights (%) in the acute oral toxicity study.

	Treatment, CFU·mL^−1^			
Organ	Acute Tocixity			
	Control	*B. amyloliquefaciens* HTI-19		
		1 × 10^9^ (LD)	3.0 × 10^9^ (MD)	1 × 10^10^ (HD)
Lung	0.53 ± 0.12	0.50 ± 0.16	0.59 ± 0.17	0.46 ± 0.23
Heart	0.39 ± 0.10	0.34 ± 0.09	0.29 ± 0.08	0.53 ± 0.35
Liver	4.20 ± 0.25	3.66 ± 0.51	3.62 ± 0.47	4.00 ± 0.73
Spleen	0.26 ± 0.04	0.25 ± 0.06	0.26 ± 0.07	0.21 ±0.05
Right kidney	0.42 ± 0.06	0.38 ± 0.08	0.39 ± 0.12	0.44 ± 0.11
Left Kidney	0.39 ± 0.05	0.37 ± 0.06	0.37 ± 0.07	0.42 ± 0.15

There were no statistical differences in each strain at the level of *p* < 0.05. Values are expressed as mean ± S.D. (*n* = 8). Statistical analysis was performed by one-way ANOVA followed by Dunnet’s t-test (LD—low dose, MD—medium dose, HD—high dose).

**Table 4:** Effects of *B. amyloliquefaciens* HTI-19 on relative organ weights (%) in the sub-acute oral toxicity study.

	Treatment, CFU·mL^−1^		
Organ	Acute Tocixity		
	Control	*B. amyloliquefaciens* HTI-19	
		1 × 10^9^ (LD)	1 × 10^10^ (HD)
Lung	0.55 ± 0.03 ^a^	0.63 ± 0.10 ^a^	0.71 ± 0.07 ^b^
Heart	0.34 ± 0.03 ^a^	0.40 ± 0.45 ^b^	0.40 ± 0.04 ^b^
Liver	4.35 ± 0.28 ^a^	4.54 ± 0.50 ^a^	4.94 ± 0.40 ^a^
Spleen	0.22 ± 0.04 ^a^	0.27 ± 0.02 ^a^	0.30 ± 0.05 ^b^
Right kidney	0.39 ± 0.04 ^a^	0.41 ± 0.04 ^a^	0.48 ± 0.10 ^a^
Left Kidney	0.36 ± 0.02 ^a^	0.39 ± 0.03 ^a^	0.45 ± 0.07 ^b^

Different superscript letters in the same row indicate statistical differences in each strain at the level of *p* < 0.05. Values are expressed as mean ± S.D. (*n* = 8). Statistical analysis was performed by one-way ANOVA followed by Dunnet’s *t*-test (LD—low dose, HD—high dose).

**Table 5:** Biochemical parameter changes following the administration of *B. amyloliquefaciens* HTI-19 in the acute toxicity study.

Parameter	Normal Range [25,26,27]	Treatment, CFU·mL^−1^			
		Acute Tocixity			
		Control	*B. amyloliquefaciens* HTI-19		
			1 × 10^9^ (LD)	3.0 × 10^9^ (MD)	1 × 10^10^ (HD)
ALP, U/L	59.00–196.00	148.20 ± 28.01 ^a^	97.63 ± 41.13 ^a^	123.13 ± 43.9 ^a^	60.83 ± 13.93 ^b^
ALT, U/L	19.20–48.70	44.00 ± 3.87	33.00 ± 10.04	44.00 ± 17.89	33.00 ± 31.26
AST, U/L	67.30–166.00	114.33 ± 14.84	132.50 ± 34.73	141.50 ± 55.91	104.17 ± 45.91
ALB, g/L	26.85–34.55	38.20 ± 2.86 ^a^	34.70 ± 9.81 ^a^	30.59 ± 8.15 ^a^	25.00 ± 7.71 ^b^
TP, g/L	49.70–73.00	71.90 ± 4.54 ^a^	63.80 ± 19.24 ^a^	58.21 ± 17.11 ^a^	44.80 ± 13.26 ^b^
Creat, umol/L	29.00–63.00	45.00 ± 2.40 ^a^	46.00 ± 11.03 ^a^	42.00 ± 11.78 ^a^	29.00 ± 6.05 ^b^

Different superscript letters in the same row indicate statistical differences in each strain at the level of *p* < 0.05. Values are expressed as mean ± SD (*n* = 8). Statistical analysis was performed by one-way ANOVA followed by Dunnet’s *t*-test (LD—low dose, MD—medium dose, HD—high dose).

**Table 6:** Effects of *B. amyloliquefaciens* HTI-19 on biochemical parameters in the subacute oral toxicity study.

Parameter	Normal Range [25,26,27]	Treatment, CFU·mL^−1^		
		Sub-Acute Tocixity		
		Control	*B. amyloliquefaciens* HTI-19	
			1 × 10^9^(LD)	1 × 10^10^ (HD)
ALP, U/L	59.00–196.00	134.33 ± 30.62	149.33 ± 15.93	153.17 ± 35.16
ALT, U/L	19.20–48.70	54.00 ± 26.43	49.00 ± 8.04	52 ± 11.47
AST, U/L	67.30–166.00	127.00 ± 52.82	117.17 ± 24.99	145.67 ± 51.64
ALB, g/L	26.85–34.55	36.8 ± 2.66	35.60 ± 1.96	38.20 ± 4.11
TBil, umol/L	1.20–8.40	3.00 ± 0.50	3.00 ± 0.59	3.00 ± 0.58
TP, g/L	49.70–73.00	71.5 ± 5.12	69.90 ± 4.00	76.0 ± 7.82
Creat, umol/L	29.00–63.00	47.00 ± 4.05	48.00 ± 3.51	46.00 ± 4.38
Urea, mmol/L	5.56–12.67	9.00 ± 1.23	8.00 ± 0.80	8.00 ± 1.90

There were no statistical differences in each strain at the level of *p* < 0.05. Values are expressed as mean ± SD (*n* = 8). Statistical analysis was performed by one-way ANOVA followed by Dunnet’s *t*-test (LD—low dose, HD—high dose).

**Table 7:** Effects of *B. amyloliquefaciens* HTI-19 on complete blood count in the subacute oral toxicity study.

Parameter	Unit	Normal Range, [25]	Treatment, CFU·mL^−1^		
			Sub-Acute Tocixity		
			Control	*B. amyloliquefaciens* HTI-19	
				1 × 10^9^(LD)	1 × 10^10^ (HD)
RBC	×10^12^/L	2.9–6.8	5.59 ± 0.90 ^a^	6.27 ± 1.20 ^a^	5.7038 ± 0.74 ^a^
Hemoglobin	g/L	86.00–153.80	136.38 ± 18.31 ^a^	151 ± 25.07 ^a^	142.38 ± 14.94 ^a^
Packed cell volume	L/L	0.10–0.47	0.38 ± 0.05 ^a^	0.39 ± 0.06^a^	0.4 ± 0.04 ^a^
Mean corpuscular volume (MCV)	fL	15.15–119.44	67.75 ± 6.38 ^a^	62.56 ± 3.23 ^a^	70.58 ± 5.33 ^a^
Mean corpuscular hemoglobin concentration (MCHC)	g/L	211.60–950.00	363.87 ± 25.41 ^a^	387.12 ± 18.34 ^a^	355.80 ± 16.26 ^a^
WBC	x10^9^/L	3.60–14.50	7.76 ± 2.54 ^a^	9.33 ± 4.11 ^a^	10.36 ± 6.75 ^a^
Neutrophils	%	13.00–61.00	12.00 ± 2.62 ^a^	14.00 ± 3.024 ^a^	18.75 ± 25.75 ^b^
Lymphocytes	%	55.00–86.00	82.50 ± 3.16 ^a^	79.50 ± 3.38 ^a^	74.9 ± 7.019 ^b^
Monocytes	%	0.00–1.00	3.75 ± 0.71 ^a^	4.625 ± 0.74 ^a^	4.50 ± 1.20 ^a^
Eosinophils	%	0.00–8.00	0.75 ± 0.46 ^a^	0.88 ± 0.835 ^a^	0.88 ± 0.35 ^a^

Different superscript letters in the same row indicate statistical differences in each strain at the level of *p* < 0.05. Values are expressed as mean ± SD *(n* = 8). Statistical analysis was performed by one-way ANOVA followed by Dunnet’s *t*-test. (LD-low dose, HD-high dose). The absence of significant changes in the vital organs such as the liver and kidney of the treated groups as observed in histopathological sections proves that the administration of *B. amyloliquefaciens* HTI-19 did not induce any anomalous lesions or inflammations.

## Data Availability

The data presented in this study are available in this article and on request from the corresponding author.
